# Adenosine and Sleep

**DOI:** 10.2174/157015909789152182

**Published:** 2009-09

**Authors:** Theresa E Bjorness, Robert W Greene

**Affiliations:** Department of Psychiatry, UTSW and Dallas VAMC, Dallas, TX, USA

## Abstract

Over the last several decades the idea that adenosine (Ado) plays a role in sleep control was postulated due in large part to pharmacological studies that showed the ability of Ado agonists to induce sleep and Ado antagonists to decrease sleep. A second wave of research involving *in vitro *cellular analytic approaches and subsequently, the use of neurochemical tools such as microdialysis, identified a population of cells within the brainstem and basal forebrain arousal centers, with activity that is both tightly coupled to thalamocortical activation and under tonic inhibitory control by Ado. Most recently, genetic tools have been used to show that Ado receptors regulate a key aspect of sleep, the slow wave activity expressed during slow wave sleep. This review will briefly introduce some of the phenomenology of sleep and then summarize the effect of Ado levels on sleep, the effect of sleep on Ado levels, and recent experiments using mutant mouse models to characterize the role for Ado in sleep control and end with a discussion of which Ado receptors are involved in such control. When taken together, these various experiments suggest that while Ado does play a role in sleep control, it is a specific role with specific functional implications and it is one of many neurotransmitters and neuromodulators affecting the complex behavior of sleep. Finally, since the majority of adenosine-related experiments in the sleep field have focused on SWS, this review will focus largely on SWS; however, the role of adenosine in REM sleep behavior will be addressed.

## PHENOMENOLOGY OF SLEEP

Sleep may be phenomenologically characterized by four criteria: a species specific posture, characteristic changes in the electroencephalogram (EEG), an increase in response threshold to environmental stimuli, and quick reversibility [76]. Using these criteria, sleep can be further divided into two main stages, slow wave sleep (SWS) and rapid eye movement sleep (REM). In mammals, EEG and electromyogram (EMG) activity is sufficient to determine sleep/waking state. During waking, the EEG is dominated by low amplitude, high frequency activity, with high muscle activity apparent in the EMG. During SWS, EEG activity is dominated by high amplitude, low frequency activity known as slow wave activity (SWA, 0.5-4.5 Hz oscillation in membrane potential), with little EMG modulation. During REM sleep, EEG activity is dominated by low amplitude, high frequency activity similar to that of waking, with EMG modulation absent except for the occasional muscle twitch. Sleep behavior is controlled by circadian and homeostatic mechanisms, with circadian rhythms influencing the time of day in which sleep expression is likely to occur and homeostatic mechanisms providing a cumulative ‘time awake’ input which results in increasing sleep drive with increasing amounts of waking. Examples of EEG traces for each of these states are shown in Fig. (**1**). The remainder of this review will focus in large part on SWS because Ado has been most closely linked, both theoretically and experimentally, to this sleep stage and to the EEG activity that characterizes it, SWA.

## BRAIN REGIONS INVOLVED IN SLEEP BEHAVIOR

The hunt to identify the brain regions sufficient and necessary for the induction and maintenance of sleep behavior has been ongoing for many years. This search has been met with limited success because while there are many brain areas that show sleep modulated activity, no single brain area is necessary for sleep. Numerous brain areas implicated in sleep control have been lesioned, however, if an animal survives, it sleeps, though sometimes sleep returns only after several weeks of recovery [38, 73]. Currently there are several brain areas that are thought to be important for sleep control, including: mesopontine tegmentum [62], hypothalamus [66], and basal forebrain [27]. *In vitro* and *in vivo* studies have determined the effect of Ado agonists and antagonists on activity within these sleep centers. In the mesopontine tegmentum, Ado agonists inhibit cholinergic neurons [50] and reduce evoked glutamatergic EPSCs and GABAergic IPSCs [2]. The mesopontine tegmentum is part of the cholinergic arousal system so decreasing activity in these cholinergic, glutamatergic, and GABAergic cells would serve to facilitate sleep and the thalamocortical neural activity of burst firing that underlies the slow wave activity in the EEG during slow wave sleep [45, 50]. In the hypothalamus, Ado agonists inhibit the wake-active hypocretin/ orexin neurons [33] and disinhibit or excite the sleep-active neurons in the preoptic/anterior hypothalamic area and ventrolateral preoptic area [11, 19, 39, 40]. Finally, in the basal forebrain, Ado agonists inhibit wake-on neurons [1, 67]. The basal forebrain has been the focus of the hypothesis that Ado is involved in sleep homeostasis due to the finding that the basal forebrain was the only cholinergic arousal center to show sustained, elevated levels of Ado after 6 hrs of sleep deprivation [44]. Nonetheless, the failure to show a similar elevation in the cholinergic brainstem center may have resulted from the small size of nuclei within the brainstem and the relatively large size of the microdialysis probe. Together, direct inhibition of wake-active neurons and their inhibition through the excitation of sleep-active neurons may increase the probability that sleep with high slow wave activity will occur. Ado may contribute to this process through inhibition of arousal centers, as well as, through inhibition of thalamocoritical systems providing excitatory drive to these same centers.

## ORIGIN OF EXTRACELLULAR ADO

Ado levels are influenced by neuronal activity. Ado is a secondary by-product of the breakdown of ATP and cAMP. When ATP is co-released with neurotransmitters, ecto-nucleotidases in the extracellular space can rapidly dephosphorylate ATP, ADP, and AMP into Ado [15]. Ado can also be released into the extracellular space by two equilibrative nucleoside transporters.

ATP release from astrocytes also contributes to extracellular levels of Ado that have a powerful modulatory effect on synaptic transmission [43]. The role of this astrocyte-derived Ado in sleep/waking homeostasis was recently investigated. Astrocytic transmitter release was prevented in a mutant mouse using a conditional knockout of the synaptobrevin II protein involved in exocytosis that was expressed only in astrocytes [22]. These mutant mice spent the same amount of time in waking, SWS, and REM sleep as wildtype mice, however mutant mice showed reduced SWA and a decrease in recovery sleep following sleep deprivation.

## INFLUENCE OF ADO LEVELS ON SLEEP AND WAKEFULNESS

In the mid to late 1900s it was found that Ado agonists decrease wakefulness and increase sleep [10, 17, 23, 46]. Furthermore, such agonists also tend to increase deeper stages of SWS at the expense of lighter SWS [49], with deep and light stages defined on the basis of amount of slow waves, greater than 50% per epoch versus less than 50% per epoch, respectively. Additionally, Ado agonists increase SWA or delta power [6, 61] as assessed by Fast Fourier Transform (FFT) analysis. SWA power reflects the relative amount of the EEG signal that falls within the SWA band (0.5-4.5 Hz).

Conversely, Ado receptor antagonists increase wakefulness and decrease sleep [61, 72, 75]. One of the most commonly used pharmacological agents, caffeine, is a nonselective Ado antagonist which primarily acts at two of the four Ado receptor subtypes, the A_1_R and A_2a_R to influence sleep/waking behavior. The estimated daily intake of caffeine in American citizens is about 280 milligrams, which is above the functional dose for decreasing sleep [29, 31]. Furthermore, caffeine and other antagonists decrease SWA within sleep as well [31, 32], an effect which is modulated by caffeine-sensitivity in humans [52]. Both agonists and antagonists affect sleep and SWA when given systemically [49, 61, 75] or within the brain [6, 39, 41, 46, 70]. Some of the biggest effects are seen when Ado is injected directly into the basal forebrain [4, 46]. This point will be discussed in detail in the next section.

In addition to agonists and antagonists, other compounds that alter endogenous Ado levels have been shown to modify sleep and SWA within sleep. These compounds include an Ado kinase inhibitor that increases Ado levels by inhibiting the phosphorylation of Ado to AMP, Ado deaminase inhibitors, which increase Ado levels by inhibiting the breakdown of Ado into inosine, and transport inhibitors, which blocks the transport of Ado into the cell. Ado kinase, deaminase, and transport inhibitors decrease wakefulness and increase sleep [45, 47, 48]. The Ado kinase inhibitor also increases SWA within SWS [47]. Furthermore, recent genetic screening experiments have shown that a genetic variant of Ado deaminase that decreases metabolism of Ado to inosine, results in an increase in deep SWS and SWA [53].

Evidence from extracellular injection of antisense mRNA is consistent with the idea that the expression of Ado receptor levels influence sleep/waking behavior. Injection of antisense deoxyoligonucleotides against Ado receptor mRNA into the basal forebrain, increases waking, while decreasing SWS and SWA [69]. Under conditions of recovery from 6 hr sleep deprivation, changes in waking, sleep, and SWA were even more pronounced. However some caution in the interpretation of these results is needed because of the well documented non-specific and “off-target” effects of antisense injections. We did not observe any change in SWS duration with localized knockout of the A_1_R [technique described in 58] in either the forebrain or the brainstem cholinergic arousal centers using an AAV-vector mediated localized expression of Cre recombinase together with a floxed A_1_R gene [unpublished observations, Ronan, P. and Greene, R.W., 2002]. Based on these observations we concluded that A_1_Rs on pre-synaptic terminals are especially important for the SWS effects of localized injections of either A_1_R agonists [46] or blockers of Ado re-uptake [45] since these pre-synaptic receptors would not be affected by localized knockout of the A_1_R gene (Ronin and Greene unpublished observations). In fact, these findings led us to use a conditional knockout of the A_1_R that would affect most of the pre-synaptic terminals that synapse onto neurons of the cholinergic arousal centers [7] as described below.

Together the evidence from Ado agonists, antagonists, an Ado kinase inhibitor, Ado deaminase inhibitors, and an Ado transport blocker all suggest that increasing Ado levels increases sleep, while decreasing Ado levels increases wakefulness.

## INFLUENCE OF BEHAVIORAL STATE (WAKEFULNESS AND SLEEP) ON ADO LEVELS WITH AN EMPHASIS ON THE BASAL FOREBRAIN

As mentioned in a previous section, injection of Ado agonists and antagonists into the brain, and specifically into the basal forebrain [46, 65], modify neural activity and ultimately influence behavioral state, arguing that modification of Ado levels is sufficient to alter sleep/waking state. This raises the question of whether sleep/waking state influences Ado levels. If Ado is, in fact, involved in controlling sleep/waking state, then Ado levels should rise during waking and decrease during subsequent sleep.

During sleep Ado levels decrease in the cortex, basal forebrain, hypothalamus, and brainstem [44, 45]. When animals are kept awake for extended periods of time (sleep deprivation), however, levels of Ado continue to rise or are stable only in one of these brain regions, the basal forebrain [4, 44]. This piece of evidence lead to a modification of the hypothesis that Ado is involved in sleep/waking control, to the hypothesis that Ado acts specifically within the basal forebrain to influence sleep/waking state. However, as previously mentioned this lack of evidence may, at least in part reflect the technical limitations of the microdialysis technique used to assess these changes. Evidence from *in vitro *studies suggests that in the brainstem cholinergic arousal center, persistent, increased synaptic glutamate release within the range of what may be expected during waking will gradually increase Ado levels in the microenvironment of the synapse and this in turn, will feedback to decrease glutamate through activation of pre-synaptic A_1_Rs [9]. This same kind of effect is likely to occur in other brain regions involved in sleep induction and maintenance and their target regions as well. Ado levels progressively rise during waking, inhibiting cholinergic and noncholinergic neurons, thereby decreasing acetylcholine release which underlies the cholinergic arousal system [50]. Furthermore, decreased acetylcholine release facilitates the transition of fast spiking to burst pause firing in thalamocortical neurons [36], which serves to synchronize neural firing, resulting in the high amplitude, low frequency activity that underlies SWA during sleep.

A recent controversy developed when Blanco-Centurion and colleagues tested the hypothesis of behavioral state modification by Ado’s action on cholinergic neurons in the basal forebrain. They selectively lesioned cholinergic basal forebrain neurons using the cholinergic-selective toxin 192 immunoglobulin-saporin (IgG-saporin) [8]. However, they found that although Ado levels did not increase in these lesioned animals, they still had normal sleep homeostasis. This finding challenged the belief that Ado modulated behavioral state by acting on cholinergic neurons in the basal forebrain. However, subsequent research using IgG-saporin has reinstated the idea that cholinergic neurons in the basal forebrain are involved in the Ado regulation of sleep homeostasis. Two groups (McCarley and Semba) directly injected IgG-saporin into the basal forebrain to destroy cholinergic neurons and found that lesioned animals displayed significantly less rebound sleep in response to acute sleep deprivation, indicating abnormal sleep homeostasis [28, 30]. Additionally, the McCarley group also used i.c.v. IgG-saporin injections to decrease basal forebrain cholinergic cell numbers and found that two weeks after injection, animals showed normal sleep homeostasis in response to sleep deprivation, while three weeks after injection, animals displayed abnormal sleep homeostasis [28]. Therefore, it is likely that Blanco-Centurion may not have found an effect of cholinergic cell lesions on sleep homeostasis because they measured the effect of sleep deprivation on homeostasis two weeks following i.c.v. injection.

## ADO RECEPTORS INVOLVED IN MODULATING SLEEP HOMEOSTASIS

One question that has fueled several recent experiments is: which Ado receptor(s) underlie the effects of Ado on sleep/waking homeostasis? There are currently 4 known Ado receptors, A_1_R, A_2a_R, A_2b_R and A_3_R. For sleep/waking homeostasis the A_1_Rs and A_2a_Rs have received the most attention due to their expression pattern in the nervous system, the availability of selective agonists and antagonists and selective molecular lesions of genes encoding the receptor subtypes. A_1_R are expressed at high levels throughout the brain, particularly in the cortex, hippocampus, thalamus and cerebellum [14, 51]. A_2a_Rs are expressed most strongly in the striatum [18]. Using RT-PCR, Dixon and colleagues found low levels of A_2a_R , A_2b_R, and A_3_R ’s expressed throughout the brain [14].

A_1_R agonists and antagonists modify sleep/waking when given peripherally [49, 72] and centrally [34, 39, 68]. A_2A_R agonists and antagonists also modify sleep/waking when given peripherally [61, 75] and centrally [12, 24, 34, 39, 56, 59]. Additionally, in humans, A_1_Rs are upregulated following sleep deprivation [16], while in rodents A_1_Rs are upregulated in the basal forebrain following sleep deprivation [3].

A_1_Rs are Gi-coupled and generally considered inhibitory, while A_2a_R s are Gs coupled and considered excitatory. A_1_Rs may modify sleep/waking by direct and indirect mechanisms including: inhibition of cholinergic neurons in the mesopontine tegmentum that are part of the cholinergic arousal system [50], inhibition wake-active hypocretin/orexin neurons in the lateral hypothalamus [33] and wake-active neurons in the basal forebrain [1, 67], and disinhibition of sleep-active neurons in the preoptic/anterior hypothalamic area and ventrolateral preoptic area [11, 40].

Mechanisms of sleep/waking homeostasis for A_2A_Rs include: inhibition of histaminergic neurons [24], excitation of sleep-active neurons in the ventrolateral preoptic nucleus [19], and modulation of acetylcholine release in the pontine reticular formation [12], resulting in increased time in SWS and REM sleep. Additionally, A_2A_R agonists increase SWS and REM sleep when injected into the prostaglandin D2 sensitive zone under the basal forebrain, while injection of A_2A_R antagonists into the same zone decreases prostaglandin D2-induced SWS [57]. The most dominant A_2a_R effect in rodents may be indirect through activation of inhibitory motor circuits in the striatum, where most of the A_2a_R s are expressed. This may explain the robust stimulant effects of the non-specific adonesine receptor antagonist, caffeine in A_1_R knock out animals, while A_2a_R knock out animals showed much subtler effects at higher doses of caffeine [25]. Locomotor activity is increased in these animals and locomotion is a powerful arousing stimulus in rodents. In contrast to the reported adenosine antagonists arousing effects being mediated through A_2a_R, injection of an A_2a_R antagonist into the ventral striatum increased time in SWS and REM sleep, as well as, decreased FOS expression within wake-active orexin neurons in the hypothalamus [55] which provides a mechanism to control sleep behavior that may not be locomotion-specific. Alternatively, this effect may have been mediated through a non-specific action of the A_2a_R antagonist to cause phosphodiesterase inhibition.

Recent experiments have used genetic manipulation to determine whether A1Rs or A2aR s are responsible for the effect of Ado on sleep/waking homeostasis. Stenberg and colleagues used constitutive A1R knockout mice and found normal sleep/homeostasis and SWA in response to sleep deprivation by gentle handling [63]. In contrast, our lab, using conditional A1R knockout mice (a Cre-loxP based system using a CAMKII promoter for the Cre recombinase gene), found a significant decrease in SWA in response to both acute and chronic sleep restriction [7]. For acute sleep restriction, we used a slowly moving treadmill (~ 3 cm/sec) to enforce waking for 4 hrs followed by 2 hrs of unrestricted behavior. For chronic sleep restriction, this 6 hr cycle (4 hrs treadmill on, 2 hrs treadmill off) was repeated 8 times across 48 hrs. The use of the treadmill to enforce waking is a potentially less variable method of sleep restriction since, unlike gentle handling, in which animals are given novel objects on an “as needed” basis in which arousal state could fluctuate, the treadmill is constantly “on” and the animals are forced to move at a relatively constant rate. Interestingly, the mice with intact A1Rs, compensated for the sleep restriction with increased SWA but unchanged SWS duration (SWS duration was compared to the same time period under non-sleep restricted conditions). The compensatory SWA response in the A1R knockout mice was largely missing. These conditional knockout mice were able to express SWA during SWS but were unable to increase SWA when sleep was restricted. Thus, the A1R receptor is involved in the homeostatic modulation of SWA, but is not necessary for SWA expression, in other words, while the A1R is not necessary for the expression of SWA (knockout mice still show EEG activity in the SWA band), the A1R does play a role in the modulation of SWA (knockout mice show significantly less sleep-deprivation induced SWA).

As for the effect of A_2a_Rs on sleep homeostasis, Urade and collegues used A_2a_R knockout mice and found a lack of modulation of sleep/waking in response to injection of A_1_R agonists into the lateral ventricle [71], suggesting that A_2a_Rs underlie the ability of Ado to modulate sleep/waking. Negative results are difficult to interpret in this case and may have resulted from an insufficient concentration of A_1_R agonist at the most relevant receptor sites, including pre-synaptic excitatory terminals onto basal forebrain neurons. In a separate report, this group showed that A_2a_R knockout mice have abnormal autonomic control during REM sleep compared to wildtype controls [54].

## A_1_R ACTIVATION AND THE FUNCTION OF SWA OF SLEEP

The loss of A_1_Rs in the CNS resulted in a remarkably specific sleep phenotype, characterized by the absence of the compensatory increase in SWA in response to sleep restriction. Although there was a significant decrease in SWA expression in A_1_R knockout mutants under baseline conditions, the effect size was small. Under baseline conditions, the mutants could perform a working memory task equally well compared to their matched wild phenotype littermates. When both groups of mice were challenged with sleep restriction, only the mutants showed a reduced performance in association with a loss of compensatory SWA response [7].

The working memory task places particular demands on prefrontal cortical circuits that involve sustained neuronal activity, thought to reflect active, short term encoding of the working memory [20]. The selective loss of compensatory SWA may have compromised the ability of the prefrontal cortical circuits to generate and sustain the needed neuronal activity for effective working memory dependent performance. This is a different kind of deficit than that involving consolidation of memory as working memory is short lasting (less than 10’s of seconds) and does not involve consolidation at all. Our findings suggest that with mild sleep restriction, working memory function may be more sensitive to compensatory SWA loss than hippocampal dependent memory and its consolidation [7].

## A_1_R ACTIVATION LEADS TO INCREASED SWA AT THE CELLULAR LEVEL IN THALAMOCORTICAL NEURONS

Several investigators have shown that modulation of A_1_Rs influence SWA without modifying sleep time. Activation of A_1_Rs can enhance SWA through two, additive mechanisms; one indirect that involves the brainstem cholinergic arousal system and the other through direct actions on thalamic and cortical neurons. Fig. (**2**) shows an illustration of both the indirect and direct sites of action and effects of Ado on SWA. The indirect Ado action results from high waking (and REM sleep) cholinergic tone in thalamocortical neurons, relative to the low tone during SWS [35, 74]. This tone is in turn modulated in the brainstem and basal forebrain cholinergic arousal centers in a state specific manner by A_1_Rs [21, 45, 50]. Acetylcholine inhibits slow frequency activity in thalamocortical neurons [13, 37, 64], so inhibition of brainstem cholinergic neurons via A_1_Rs during SWS allows the expression of slow wave activity.

**Fig. (3):**
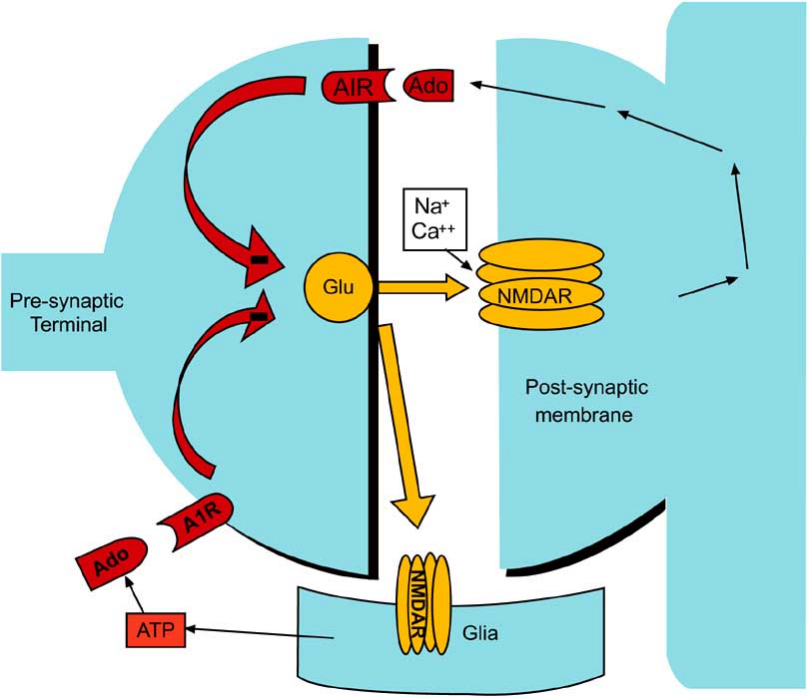
Pre-synaptic effect of adenosine on glutamatergic activity. During cellular activity, adenosine is released from neurons and glia. This adenosine feeds back onto pre-synaptic neurons to inhibit glutamate release *via* A_1_Rs. This inhibition of an excitatory compound reduces neural activity.

The direct effect of A_1_R activation to facilitate SWA of thalamic and cortical neurons is due to a combination of increased GIRK channel conductance and decreased hyperpolarization activated current and a relative functional deafferentation resulting from pre-synaptic inhibition (as shown in Fig. **3**). *In vitro* circuit analysis has shown that Ado will enhance slow oscillations of single neurons in the absence of other modulatory input [42]. Localized increase of Ado can induce these post-synaptic effects and when combined with local pre-synaptic A_1_R activation to reduce afferent input, and can increase SWA measured with local field recordings as demonstrated by localized blockade of Ado uptake with nitrobenzylthioinoside [45]. It is reasonable to suggest that, just as with brainstem arousal neurons [9], increased synaptic activation of NMDARs during waking in the thalamocortical system will slowly increase local Ado concentration to directly enhance SWA in localized thalamocortical circuits. In fact asymmetric increases in SWA activity have been observed in association with asymmetric use-dependent activity during waking [26].

The modulation of SWA in a use-dependent fashion is a key prediction of the hypothesis that sleep serves as a restorative function for the brain. Benington and Heller posited that the increased release of adenosine that occurs due to waking activity, works through A_1_Rs to facilitate the expression of SWA, which allows for the glycogen stores, depleted during waking, to be replenished during sleep [5]. Recently Pack and colleagues reviewed research that supports the overall hypothesis and have included additional metabolic-factors, besides Ado, that may play a role in energy restoration during sleep [60]. Finally, the use of A_1_R knockout animals that show reduced SWA following sleep deprivation [7] and the use of a mutant mouse with inhibited astrocytic-release of adenosine [22] indicate that adenosine is an important factor controlling SWA expression. Additionally, these studies show that increased SWA expression during recovery sleep following sleep deprivation is important for cognitive performance [7], but increased SWA pressure during waking impairs recognition memory [22].

## CONCLUDING REMARKS

There is ample evidence that Ado plays a role in regulating sleep/waking behavior, as well as, SWA. Together this evidence suggests that both A_1_Rs and A_2a_Rs act in various areas of the brain to decrease neural activity and facilitate sleep, although the role of the A_2a_R may be indirect via the striatal locomotor systems. A_1_Rs primarily act to both reduce cholinergic tone and, in thalamocortical systems, to facilitate cellular oscillations at SWA frequency in response to sleep loss. Gene deletion of CNS A_1_Rs prevents the compensatory SWA enhancement when sleep is restricted, in association with reduced cognitive performance. This suggests a functional role for A_1_R mediated compensatory SWA activity to maintain normal cognitive performance.

## Figures and Tables

**Fig. (1):**
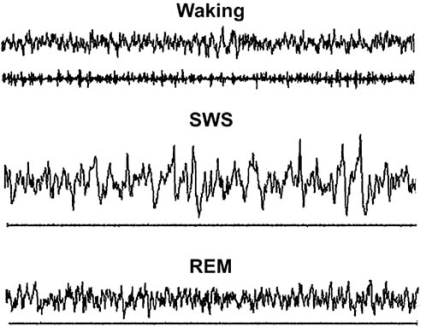
Raw electroencephalograph and electromyography examples of waking, SWS, and REM sleep from a C57/BL6 mouse. EEG signals were recorded from screw electrodes overlying the cortex, while EMG signals were recorded from panel electrodes from the dorsal neck muscle. Each trace shows a 10 sec epoch. The y-axis is set at 250 µv.

**Fig. (2):**
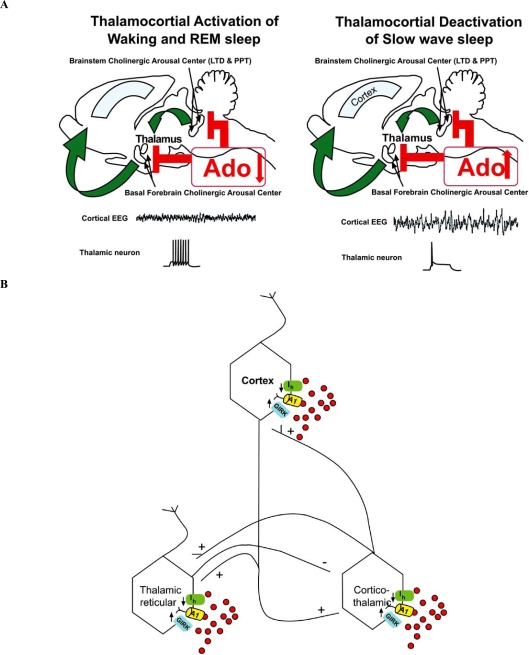
**A**. Indirect A_1_R -mediated modulation SWA. Projections from the brainstem reticular formation cholinergic arousal center to the thalamus and from basal forebrain cholinergic arousal center to the cortex and thalamus mediate the cholinergic tone. During waking, cholinergic tone is high, resulting in a desynchronized, high frequency cortical EEG (middle trace), as is monoaminergic tone (not shown). During REM sleep only cholinergic tone is high in association with a desynchronized EEG, similar to that observed during waking. The desynchronized activity is due in part to the cholinergically induced depolarization of thalamic and cortical neurons facilitating synaptically responsive non-bursting spiking patterns (bottom trace). During SWS, cholinergic tone is decreased due, in part to increased adenosine mediated inhibition of cholinergic center arousal neurons. The emergence of synchronized, low frequency cortical EEG (middle trace) requires this decreased activity in the cholinergic arousal centers of the forebrain and brainstem, allowing hyperpolarization of thalamic and cortical neurons. This is a necessary condition for burst-pause oscillatory firing of thalamic and cortical neurons (bottom trace). The thalamic neuron recordings were made *in vitro*, under control, waking-like conditions (bottom left) and in the presence of adenosine to approximate SWS-like conditions (modified with permission from [42]). **B**. Activation of A_1_Rs can directly facilitate SWA. Ado hyperpolarizes thalamic and cortical neurons by increasing potassium conductance through the GIRK channel and by decreasing the Ih current, which facilitates burst-pause firing at the expense of single spikes [42]. Both these changes also reduce cell conductance at the threshold for burst generation providing an additional facilatory effect on bursting. These effects may be synchronized by the thalamocortical- thalamo-reticular circuits to generate the high amplitude SWA observed during sleep.
